# Integrating cannabis and gaming: re-evaluating their business intersection

**DOI:** 10.1186/s42238-025-00382-9

**Published:** 2026-02-09

**Authors:** Riana Durrett, Juliana Ness, Marla Royne Stafford, Margaret McLetchie, Jordyn Sanders

**Affiliations:** 1 Cannabis Policy Institute, University of Nevada-Las Vegas, Las Vegas, NV 89154 USA; 2 Cannabis Compliance Board, State of Nevada, Nevada Carson City, USA; 3 William S. Boyd School of Law, University of Nevada-Las Vegas, Las Vegas, NV 89154 USA; 4 Department of Marketing & International Business, University of Nevada-Las Vegas, Las Vegas, NV 89154 USA; 5 McLetchie Law, Las Vegas, NV 89101 USA

## Abstract

This paper examines the intersection of cannabis and gaming laws in the United States. A particular emphasis is placed on Nevada for its recognition as a leader in the gaming and cannabis industry. To better understand the issue, we explored various factors contributing to the separation, ranging from federal to local concerns at both the operational and state levels. Given the unpredictability of federal enforcement, gaming regulators and state regulators must address the risks associated with cannabis innovation. Despite the exclusion, there is growing support for incorporating cannabis into the gaming sector, similar to other markets, particularly in high-profile areas like the Las Vegas Strip. The paper questions whether the separations are appropriate within legal, policy, and business structures, apart from the activities. While acknowledging that public initiatives focused on practical activities are essential, this paper focuses on the framework that led to the original findings. Moreover, this paper examines the regulatory differences between jurisdictions that permit gambling and gaming, and identifies potential risks associated with the separation of these industries.

## Introduction

Because the gaming industry emerged from a background similar to cannabis, once prohibited, widely used, and eventually brought under regulatory oversight, it might be expected that the two sectors would develop together. Yet when states began legalizing medical and adult-use cannabis, regulators moved quickly to create clear boundaries between cannabis and gaming. In Nevada, for example, gaming regulators emphasized that federal cannabis prohibition prevented gaming licensees from participating in, hosting, or investing in cannabis businesses (Valley 2017). Cannabis sales are prohibited on gaming premises, and the two activities cannot be colocated. Similar regulatory separations appear in other states, including restrictions that prevent gaming licensees from investing in cannabis operations. Notably, Colorado initially adopted a Nevada-style separation but later changed its position, permitting gaming licensees to hold cannabis licenses (CO S.B. 19–224, 2019); Ballard Spahr 2018).

Nevada’s approach remains uniquely explicit. Its regulatory structure creates a strong and formal separation between the industries. Because gaming operators often hold licenses in multiple states, decisions in one jurisdiction can affect suitability in others. For instance, if a gaming company operating in New Jersey allowed cannabis activity at its Atlantic City property, that company could jeopardize its Nevada license due to cannabis activity’s violation of federal law. Nevada regulators could then determine that the operator is unsuitable for licensure under Nevada law (NRS § 463.720, (2023)). Multi-jurisdictional licensing, therefore, magnifies the consequences of any deviation from Nevada’s strict separation rules.

At the time this paper was drafted, no state, province, or tribal jurisdiction in the United States or Canada had authorized colocated gaming and cannabis businesses. Only Colorado formally allows a gaming license holder to obtain a cannabis license. Even in Colorado, no operators have chosen to do so, likely due to the illegality of federal regulations and reputational considerations. The interconnected nature of gaming regulation reinforces this hesitation. Colorado, Rhode Island, New Jersey, and Massachusetts authorize regulators to consider disciplinary actions or violations in other jurisdictions when evaluating suitability (CRS § 44–30–505, 2023); MA Gen. Laws ch. 23K § 12 l, 2023); N.J. Stat. Ann. § 5:12–86, (N.J. 2024)). Rhode Island once included similar statutory language. Although it was repealed, the underlying principle remains embedded in regulatory practice through background checks and suitability reviews (230-RICR-30–30-2, 2022).

The purpose of this paper is to reexamine whether the longstanding separation between gaming and cannabis remains justified in light of evolving legal frameworks, changes in federal enforcement priorities, and rapidly shifting public attitudes toward cannabis. To guide this analysis, we pose the following research question: Do the legal and regulatory conditions that initially justified the mandatory separation between gaming and cannabis, including concerns about federal illegality, multi-jurisdictional licensing, and reputational risk, remain persuasive in light of current developments in cannabis policy and public opinion?

The paper proceeds as follows. We first provide background on Nevada’s state, local, and regulatory prohibitions on gaming licensees engaging in cannabis business activity. We then analyze the reasons originally cited for these separations and evaluate whether shifts in federal policy, enforcement posture, and public opinion alter the rationale for maintaining strict barriers. Next, we examine how jurisdictions with legal gaming and adult-use cannabis, including Colorado, New Jersey, Rhode Island, Maryland, and a sovereign tribal nation, structure the relationship between the two industries. The paper concludes with a discussion of issues policymakers will need to address should future changes in the law make integration a realistic option.

It is important to note that the original purpose of these separations was not grounded in public health or consumer protection. The separations were justified as necessary to protect gaming operators from federal law enforcement and reputational harm. Public health concerns surrounding cannabis use and gambling are legitimate and relevant to broader policy debates, but they were not the basis for the regulatory barriers examined here. Nevertheless, public health considerations arise in related contexts, including restrictions on cannabis consumption in bars, restaurants, and university housing, and these comparisons provide additional perspective on how cannabis is regulated in shared or entertainment-oriented environments. Moreover, more recent work has examined newer work on harms to “affected others,” in the gambling environment (Dowling et al. 2025), on gambling harm for family members and communities.

Moreover, the absence of regulated, colocated spaces still produces public health implications. For example, prohibiting consumption in licensed venues may push cannabis use into private or unregulated environments with fewer safeguards for vulnerable populations (Jameson, Liu, and Mitchell, 2024). Additionally, some distance requirements apply equally to medical cannabis businesses, raising questions about why medical cannabis is treated differently from other controlled substances. Pharmacies are not subject to similar distance requirements from casinos, even though they dispense medications that can impair cognition.

While this paper does not advocate integration, it acknowledges that consumers already privately combine cannabis and gaming, particularly given the growth of online gaming and broad access to legal cannabis. The central question, therefore, is not whether integration should occur, but whether the original legal and regulatory reasons for prohibiting it remain persuasive in the rapidly changing policy landscape surrounding cannabis.

## Nevada’s gaming and cannabis separations

### Why Nevada is important to understanding gaming in general

This paper examines the separation between cannabis and gaming business activities across several jurisdictions, including Nevada, Canada, tribal nations, New Jersey, and Colorado. Because gaming is a multi-jurisdictional industry and regulatory decisions in one state can affect suitability in others, an analysis of these intersections requires attention to the frameworks that shape licensing across state and tribal boundaries. Nevada serves as the central case study for this paper because it has the most explicit regulatory separation between gaming and cannabis in the United States and because it remains a national leader in gaming law and regulatory design (Faiss and Gemignani 2011a, b).

Nevada’s stature is the result of its long history as the first state to legalize commercial gaming in a structured and durable manner. Its licensing processes, which include extensive background investigations, financial disclosures, and suitability standards, have provided a template for other states to develop or modernize their own regulatory systems. For example, New Jersey adopted similarly comprehensive licensing procedures to ensure the integrity of its gaming sector (Bryan RH 2020). Colorado also initially aligned its regulatory approach with Nevada by prohibiting the intersection of cannabis and gaming (Ballard Spahr 2018). This early alignment highlights the enduring impact of Nevada’s regulatory model on the nation.

Although Nevada is the focal point of this analysis, contemporary gaming is not confined to any single jurisdiction. Many operators hold licenses in multiple states, and some also operate on tribal lands or in international markets. As a result, actions taken in one jurisdiction may create licensing risks in other jurisdictions. Nevada’s well-established position in the gaming industry, combined with its detailed statutory and regulatory separation from cannabis, provides an instructive lens through which to evaluate the original justifications for separating the two activities and assess whether those justifications remain persuasive today.

### Nevada’s laws & regulations establishing separation between cannabis & gaming

Section 678B of the Nevada Revised Statutes requires a 1,500-foot physical separation between any cannabis facility, whether medical ((NRS) Nev. Rev. Stat. Ann 678B.250(3)(a)(2)(II), 2019; NRS 678B.210, 2019) or adult-use (NRS 678B.250, 2019), and any licensed gaming establishment. Nevada also maintains a regulatory separation that prohibits gaming licensees from engaging in business activities with licensed cannabis establishments. Clark County reinforces this separation through Ordinance 8.65.140, which prohibits cannabis delivery to any restricted or nonrestricted gaming establishment, including those located along the Las Vegas Strip (Clark County, (Clark 2020)).

Although the Nevada Cannabis Compliance Board was modeled in part on the Nevada Gaming Control Board, a strict separation between the gaming and cannabis industries remains in place (Sisolak 2019). In 2014, the Nevada Gaming Control Board issued a formal notice advising licensees to avoid any investment or association with cannabis businesses because cannabis remains illegal under federal law (Johnson 2014). Gaming operators are required to comply with all local, state, and federal laws, and violations may place their gaming licenses at risk (NRS 463.720, 2023). The notice concluded that any involvement with cannabis could reflect discredit upon gaming in the State of Nevada, consistent with the public policy objectives outlined in NRS 463.0129.

Both the Nevada Gaming Commission and the Gaming Policy Committee have reinforced this legal and regulatory boundary. Commissioners have emphasized that the reputation of the gaming industry is highly sensitive and that operators must maintain a clear separation from activities involving substances classified as illegal under federal law (Velotta 2017). One commissioner stated that short-term financial gains would not outweigh the damage that association with cannabis could cause to the industry's integrity and the state's reputation (O'Neal N. 2017).

The Gaming Policy Committee adopted a resolution recommending that Nevada gaming licensees refrain from participating in the cannabis industry (GPC, 2018). The resolution permitted cannabis conventions and trade shows at gaming establishments but otherwise clarified that licensees should not contract with, lease property to, maintain business relationships with, or provide financing to cannabis businesses (GPC, 2018; Segerblom, 2020). The Committee cited Nevada Gaming Commission Regulation 5.011 (n.d), which identifies grounds for disciplinary action, to underscore the seriousness of maintaining this separation. The formal adoption of the resolution was announced to the industry on May 4, 2018 (Bell 2018).

In 2019, Assembly Bill 533 amended Nevada law by creating the Cannabis Compliance Board and codifying the physical separation between cannabis and gaming establishments (Governor Sisolak 2019; Nevada Legislature 2019a; Nevada 2019b). The bill incorporated the 1,500-foot buffer into statute, a change welcomed by the gaming industry due to ongoing concerns related to the federal status of cannabis (Yeager and Gibson 2019). For context, cannabis establishments are also required to be located at least 1,000 feet from public or private schools (NRS 678B.210(3)(a)(2)(II) ((2019)).

### Legal and regulatory reasons for the separation of the two industries

While the original rationale for prohibiting gaming licensees from associating with cannabis businesses reflected legitimate concerns at the time, particularly regarding the protection of a high-revenue and highly regulated industry, the evolution of cannabis laws and enforcement priorities now raises questions about whether those early decisions should be revisited. The Nevada Gaming Control Board’s 2014 notice, which instructed licensees to avoid participation in state legal cannabis activities, is now a decade old and was developed under considerably different legal and policy conditions. The prohibition rested on three primary concerns. First, cannabis was and remains classified as a Schedule I substance under federal law, creating potential enforcement risks for businesses associated with the industry. Second, cannabis operators historically faced limited access to banking and financial services that comply with federal guidelines, which could complicate the financial integrity obligations imposed on gaming licensees. Third, regulators feared that any association with cannabis might harm the integrity, reputation, and public image of Nevada’s gaming industry. These considerations shaped the initial regulatory wall between the two sectors and continue to influence the broader discussion about whether the separation remains justified today.

## Do the reasons cited for separating the industries persist?

### Federal enforcement and Schedule I status

A significant reason for maintaining a separation between the cannabis and gaming industries is the requirement that gaming licensees comply with all federal, state, and local laws. Nevada Gaming Commission Regulation 5.011 (n.d) requires licensees to comply with applicable laws and regulations, and violations can result in disciplinary action, including suspension or revocation of a license (NRS 678D.510(2)(c) 2021; NGC, n.d.). Because cannabis remains prohibited under federal law, any association with state legal cannabis activity theoretically exposes gaming licensees to federal risk.

A gaming license is a revocable privilege in every primary gaming jurisdiction, including Nevada, Colorado, and New Jersey (NRS 463.0129(2), (2024); Colo. Rev. Stat. § 44–30–102(d), 2023); N.J.S.A. 5:12–1(8), 2024). These licenses support industries that generate substantial revenue. Nevada alone reported more than $ 15 billion in gross gaming revenue in 2023, and states such as Colorado, New Jersey, and Maryland each reported more than $1 billion (American Gaming Association, 2024). Because the loss of a gaming license would effectively eliminate a company’s ability to operate, licensees avoid activities that may pose any threat to license security.

For that reason, many operators view involvement with the cannabis industry as an unnecessary and unpredictable risk. Representatives from the gaming industry have argued that the possible financial return from cannabis business activities does not justify the danger of losing a gaming license if the federal government were to enforce federal cannabis laws against operators or properties associated with the activity (Komenda 2019).

Although gaming companies do not hold a federal gaming license and therefore cannot lose licensure at the federal level, they still face federal obligations. Gaming companies are considered financial institutions under the Bank Secrecy Act, and involvement with an industry that has historically operated without access to federally compliant banking may raise concerns about compliance with anti-money laundering rules (Bank Secrecy Act 1970). Nevada also has a historical memory of narrowly avoiding federal intervention that could have damaged its gaming industry (Faiss and Gemignani 2011a, b). Due to this history, regulators and operators may continue to adopt a conservative approach when evaluating risks associated with any federally prohibited activity.

At the same time, scholars note that the federal government has taken a restrained approach toward state legal cannabis markets for more than a decade. Some argue that the fear of federal enforcement may function less as an imminent legal threat and more as a justification that allows states to preserve their existing regulatory structures and economic interests (Mikos 2021). From this perspective, the persistence of federal enforcement concerns may reflect a combination of risk aversion, historical regulatory culture, and strategic policymaking rather than a clear likelihood of federal intervention.

### Federal enforcement developments

Federal attitudes toward cannabis enforcement have shifted substantially in recent years, and these developments are relevant when assessing whether the original separation between gaming and cannabis remains justified. In October 2022, President Biden directed the Department of Health and Human Services to review the scheduling of cannabis under the Controlled Substances Act (Chemerinsky E 2015). In response, the Department recommended in 2024 that the Drug Enforcement Administration move cannabis from Schedule I to Schedule III, in recognition of accepted medical use and reduced abuse potential (Federal Register 2024). Public comments also show strong support for rescheduling. Data collected by Headset indicate that 92.45% of public comments submitted during the DEA’s rescheduling period favored reclassification, while 7.55% opposed it (Malyshev and Ganley 2024). This shift in public sentiment is relevant to the broader evaluation of the separation between cannabis and gaming.

The United States Supreme Court has also acknowledged inconsistencies in federal cannabis enforcement. Justice Clarence Thomas noted that the federal government’s approach to cannabis is internally contradictory, describing it as a “half in, half out” system that undermines the logic of continued prohibition of intrastate cannabis activity (Thomas, 2021). He further suggested that the current federal posture is difficult to reconcile with ongoing non-enforcement and may no longer represent a coherent or sustainable federal policy. These observations, although not binding legal conclusions, reflect a broader recognition that federal enforcement has diminished in practice, and that federal law no longer functions in a manner consistent with the realities of state regulation.

Federal cannabis policy remains subject to ongoing political uncertainty. President Trump has made statements supportive of cannabis reform, yet several of his recent appointments have held opposing views (Jaeger 2025a, b). Even with federal leadership supporting and directing rescheduling, several challenges remain, including the United States’ obligations under international drug control treaties, the need to develop a federal regulatory structure, and uncertainty surrounding the division of authority between federal and state governments.

Although federal enforcement has generally retreated, a few agencies continue to enforce federal cannabis laws in ways that can create uncertainty. In 2024, United States Customs and Border Protection in New Mexico seized shipments of state-licensed cannabis at internal border checkpoints (Fertig 2024; Jaeger 2024a, b). These actions represent a departure from the prevailing federal practice of avoiding interference with cannabis activities conducted in compliance with state law. CBP officials emphasized the continuing federal illegality of cannabis, even as other federal agencies have taken a more restrained approach (Castañeda 2022); Lee 2024). Importantly, these seizures did not result in criminal charges and appear to be limited to specific local enforcement decisions, rather than a national shift in enforcement policy. Outside of such isolated incidents, most federal enforcement has focused on unlicensed operations or activities that violate state regulations (Sacco et al. 2024; Chemerinsky E 2015).

In summary, the federal government’s approach to cannabis has evolved significantly over the past decade. Although political leadership and administrative priorities can influence the pace of reform, the broader trend suggests a shift toward reduced federal enforcement. The appointment of former DEA agent Derek Maltz has raised concerns among rescheduling advocates due to his opposition to cannabis reform (Jaeger 2025a, b). Yet, political statements continue to suggest support for revisiting cannabis’s federal status (Roberts 2024). Even when cannabis is reclassified to Schedule III, recreational cannabis activity would remain prohibited under federal law, and gaming regulators may still perceive a risk associated with involvement in the cannabis industry. Nonetheless, as legal scholars have observed, fears of a federal crackdown on state-legal cannabis markets appear overstated, given the federal government’s pattern of non-interference (Mikos 2021). These developments raise legitimate questions about whether federal enforcement concerns should continue to serve as a primary justification for separating cannabis and gaming activities.

### Financial enforcement & the lack of banking options

Under the Bank Secrecy Act, casinos with gross gaming revenues exceeding $1 million are classified as financial institutions. They are required to maintain rigorous customer identification, transaction monitoring, record-keeping, and suspicious activity reporting procedures (IRS, 2023). These requirements have produced a highly structured compliance framework intended to prevent money laundering and other illicit financial activities. Because casinos operate under heightened federal scrutiny, their existing anti-money laundering controls are often cited as a barrier to participating in cannabis related business activities, particularly where cannabis transactions remain cash-intensive.

If Nevada were to reconsider the existing prohibitions on interaction between gaming and cannabis businesses, several financial considerations would require close evaluation. First, guidance issued by the Financial Crimes Enforcement Network in 2014 outlines how banks may work with cannabis businesses while still complying with Bank Secrecy Act obligations (FinCEN, 2014). Currency Transaction Reports and Suspicious Activity Reports remain mandatory, but cannabis related reports are categorized separately, which provides a structured pathway for banks that choose to serve cannabis clients. This demonstrates that participation in federally compliant financial oversight is not precluded simply by the presence of cannabis related transactions. Second, despite the compliance burdens, an increasing number of financial institutions are opting to work with cannabis businesses. In 2024, 505 banks and 178 credit unions filed cannabis related Suspicious Activity Reports, indicating that many institutions have developed procedures that allow them to engage with the cannabis sector while maintaining federal compliance (FinCEN, 2024). These trends suggest that the existence of federal reporting requirements has not prevented financial institutions from entering the market.

The American Gaming Association has requested clarification on how FinCEN’s 2014 guidance applies in the gaming context, particularly regarding concerns that individuals involved with cannabis businesses might attempt to use casinos as intermediaries for depositing funds into the banking system (Fuerszt, Ittleman, and Joseph, 2016). In response to these concerns, the Association incorporated provisions addressing patrons with cannabis-related ties into its 2019 and 2020 Best Practices for Money Laundering Compliance (AGA, (2019-2024) 2019; Wolf 2020). These developments highlight the extent to which casinos already operate within a robust compliance environment that is capable of identifying and monitoring high-risk financial activities.

Some scholars also note that state-regulated cannabis businesses have demonstrated increasing financial transparency and are no longer the cash-only operations they were often assumed to be during the early years of legalization (El Hilali E 2024). Given the evolution of compliance practices in both industries, casinos may be better positioned than initially assumed to manage interactions with cannabis related entities without compromising regulatory integrity (Alessa 2021). These financial developments do not resolve the separation issue, but they illustrate that the original concerns about anti-money laundering risks may warrant reassessment in light of current regulatory capabilities.

### The potential negative impact on gaming’s integrity, reputation, and image

#### Integrity

While there may be pathways to allow interaction between the gaming and cannabis industries legally, maintaining the integrity of the gaming industry remains a central priority, particularly in Nevada (NRS 463.0129, 2024). Integrity creates a legal obligation for gaming regulators to ensure trust and confidence in the gaming sector. Reputation, by contrast, refers to the industry’s public image. Both concepts are important for effective gaming regulation. Still, integrity carries statutory weight and therefore plays a more significant role in shaping regulatory decisions, whereas reputation functions more as a communication and marketing concern.

When Nevada legalized cannabis, stakeholders expressed concern that its association with gaming could jeopardize gaming integrity (Bell 2018; Valley 2017). Nevada law emphasizes that gaming must be free of criminal or corruptive influences (NRS 463.0129, 2024). Because cannabis remains criminalized under federal law, regulators have viewed any potential interaction between the two industries as creating risk for operators who must demonstrate compliance with all applicable laws. This concern continues to be one of the primary reasons cited for maintaining the separation between gaming and cannabis.

At the same time, several developments complicate the assumption that federal illegality necessarily threatens gaming integrity. Federal guidance, such as the 2013 Cole Memorandum, which indicated non-interference with state-regulated cannabis markets, and more recent federal actions supporting a review of cannabis scheduling, suggest that the practical risk of enforcement may be limited (U.S. Department of Justice 2013; Roberts 2024). These developments raise the question of whether the integrity of gaming would be compromised if cannabis businesses were held to compliance standards similar to those applied to gaming, such as mandatory customer identification and reporting requirements. Such measures could both enhance transparency and impose new burdens, and their impact on cannabis sales and operations would require careful evaluation.

#### Reputation and image

Reputation concerns the overall public image and perception of the gaming industry. As Nevada’s cannabis industry began to develop, regulators expressed concern that association with cannabis could harm the reputation of Nevada's gaming industry. Former Nevada Gaming Commission Chair Tony Alamo stated that involvement with cannabis business activities “would tend to reflect discredit upon gaming in the State of Nevada” (Valley 2017). The emphasis on reputation aligns with the industry’s long-standing commitment to presenting an image of honesty and world-class entertainment.

However, public attitudes toward cannabis have changed substantially since Nevada regulators first articulated these concerns. When the Nevada Gaming Control Board issued its notice advising licensees to avoid cannabis business activities, only four states had legalized adult-use cannabis, and twenty-four allowed medical use (MJBizDaily 2024). By 2018, when the Gaming Policy Committee issued its resolution reinforcing the separation, ten states had legalized adult use, and thirty-six had legalized medical programs (MJBizDaily 2024). As of 2025, twenty-four states allow adult recreational use and forty provide legal access to medical cannabis (MJBizDaily 2024; DISA 2025). Approximately seventy-four percent of Americans live in a state with either medical or adult-use cannabis, and more than half live in a state with legalized recreational use (Chapekis, 2024). Several additional states are actively considering legalization in 2025, including Wisconsin, Pennsylvania, and Kansas (Levenson 2025; Jaeger 2024a, b; Lange 2025; Caruso S 2025; Mesa 2025).

Cannabis consumption patterns have also shifted. Roughly seventy-two percent of Americans report having used cannabis at some point in their lives (DiBella 2024). These changes in public acceptance have been acknowledged at the federal level, including in President Biden’s 2024 State of the Union address, which referenced the need to reassess cannabis scheduling (Biden 2024). It is unlikely that early regulatory statements anticipated the scale of public support that has since emerged.

As public attitudes have shifted, concerns that cannabis activity would damage the reputation of gaming warrant reexamination. Research indicates that interest in cannabis related activities during travel is growing. A 2020 study by MMGY Travel Intelligence and Enlighten Strategies found that 29% of active leisure travelers and 18% of all Americans expressed interest in cannabis activities while on vacation (Turner 2020). Cannabis tourism has grown into a significant market, with an estimated valuation of approximately $17 billion in the United States (Yakowicz 2022). Colorado reports more than one billion dollars annually in cannabis tourism revenue (Bock I 2023). In Nevada, the presence of the world’s largest dispensary in Las Vegas underscores the city’s growing reputation as a destination for cannabis consumers (Vij 2023; Bock I 2023). Some analysts now describe Nevada’s regulatory model for cannabis as highly structured and among the more comprehensive in the country (McBride 2024; Rodgers 2019), paralleling the state’s established reputation in gaming regulation (Silver 2018).

These developments do not eliminate reputation concerns, but they suggest that public perception of cannabis has changed in ways that differ significantly from the environment in which earlier regulatory prohibitions were adopted. As a jurisdiction known for regulatory leadership, Nevada is a logical setting for reassessing how shifting public attitudes may affect the rationale for maintaining a strict separation between gaming and cannabis.

### Public health reasons for the separation of the two activities

Public health considerations, including the potential effects of co-consuming cannabis and engaging in gaming activities, represent legitimate concerns that warrant ongoing research and careful policy attention. In recent years, gambling has been increasingly framed as a public health issue, with scholarship documenting links to addiction, mental health challenges, financial strain, relationship disruption, and broader social harms that extend beyond clinically defined “problem gambling” (e.g., Blaszczynski et al. 2011; Langham et al. 2016; Wardle et al. 2024). Conceptual work on gambling-related harm emphasizes that adverse consequences are experienced not only by gamblers themselves but also by affected others and communities, and these harms are shaped by product design, availability, and regulatory context rather than individual behavior alone (Langham et al. 2016; Wardle et al. 2024; Reynolds et al. 2020).

A parallel public health discourse has emerged around cannabis. Reviews of the impact of legalizing recreational cannabis in several U.S. states highlight increased availability and potency, shifts in patterns of heavy or chronic use, and measurable changes in outcomes such as emergency department presentations and cannabis-related hospitalizations (Hall & Lynskey 2016). Clinical and epidemiological evidence also points to risks related to cognitive impairment, mental health, and vulnerable populations, particularly when use begins early or is frequent and sustained (Volkow et al. 2014). Taken together, these bodies of work suggest that the intersection of cannabis use and gambling participation raises plausible concerns about cumulative or interacting harms, even if the empirical evidence on co-use remains limited.

At the same time, public health considerations were not the explicit basis for Nevada’s original decision to maintain a strict separation between cannabis and gaming. The regulatory architecture was grounded primarily in concerns about federal illegality, financial compliance, and the protection of gaming integrity and reputation. Accordingly, this article focuses on the legal, regulatory, and policy structures that continue to maintain that separation and evaluates whether the stated rationales remain justified in light of evolving cannabis laws, shifts in public perception, and changes in federal enforcement practices. Assessing the health consequences of cannabis use, gambling, or their potential co-use—while clearly crucial for public health, consumer protection, and responsible gambling policy—falls outside the scope of the present analysis and is identified instead as a critical agenda for future interdisciplinary research.

## Other jurisdictions

One interesting dynamic among jurisdictions with legal gaming and legal cannabis is that the rules around cannabis and gaming integration have not been expressly adopted in many jurisdictions, but there could be unspoken separations. It is likely understood in jurisdictions outside Nevada that collocating a gaming operation and a cannabis operation would impact a gaming operation’s license in Nevada, which is a sufficient threat to avoid such activity. However, it is unclear whether jurisdictions outside Nevada (other than Colorado) allow investment in both gaming and cannabis. It is also unclear if regulatory agencies outside Nevada monitor this. This warrants further evaluation. Thus, we offer a discussion on existing laws related to cannabis and gaming integration outside of Nevada. A comparison of the different jurisdictions is presented in Table 1.

**Table 1:** A Comparative Overview of State Cannabis and Gaming Regulations

Jurisdiction	Legal Gaming	Legal Cannabis (Recreational)	Is there a prohibition on simultaneous investment in both industries?	Is there any law that expressly separates the cannabis and gaming industries?	How does the law specifically distinguish between the sectors?
NV	Yes Under NRS Chapter 463	Yes Under NRS 678D	Yes See Notice # 2018–39 from the Nevada Gaming Policy Committee	Yes	State Statute: Ex: NRS 678B.210(3)(a)(2)(II) and NRS 678B.210(3)(a)(2)(II) Local Ordinances: Ex. Clark County Ordinance 8.65.140 Delivery of products to consumers
CO	Yes Under (CRS CRS 44–30)	Yes Under CRS (CRS 44–10)	No	No	Colorado allows gaming licensees to hold cannabis licenses (and vice-versa) through CO SB 19–244
NJ	Yes, under N.J. Stat. § 5:12–119	Yes, under N.J.A.C. 17:30 ((N.J.A.C. 2023))	No no guidance or regulations prohibiting simultaneous investment	Yes	The two industries are separated through Public Law: 2021 c.025 Assembly, No. 5342, which is the prohibition of smoking/vaping cannabis in casinos. However, cannabis has been rumored to assist in revitalizing the Atlantic City Boardwalk, known for its gaming
RI	Yes, under RI Gen. Laws § 42–61.2–3	Yes, under R.I. Gen. Laws § 21–28.(The 11–29)	No, There is no direct regulatory or statutory prohibition on investment	No	The prohibition of cannabis in casinos is operator-based rather than governmentally enforced. Operators even allow cannabis smoking outside of the casino (potentially on the property)
MD	Yes, under Md. Code Ann., State Gov't § 9-1A-01 (2008)	Yes, under Md. Code Ann., Alc. Bev. § 36–201 (2023)	No direct ban on investment	No	Under Md. Code Ann., Alcoholic Beverages and Cannabis § 9–2705 ((Md. 2021)), no cannabis licensee can have illegal gaming on-site
Tribes	Potentially, But it depends on the Tribal compact. So far, no tribal contracts specifically allow cannabis casinos	Potentially, But it depends on the location of the tribe, the state laws on cannabis, and any compacts regarding cannabis on tribal lands	No, But it depends on the Tribe; NIGC allows simultaneous investment in cannabis and gaming if the tribe enables it	Yes	NIGC released a bulletin in 2021 detailing cannabis and gaming. The bulletin left most decisions to the Tribe but included limitations, such as prohibiting cannabis sales within gaming institutions

### Colorado

Colorado occupies a unique position in that it expressly allows individuals or entities to hold both cannabis and gaming licenses. This policy emerged from recommendations in the 2018 sunset review by the Department of Regulatory Agencies. It was incorporated into Senate Bill 19–224, which aimed to update and strengthen the state’s regulated marijuana programs (Colorado Legislature 2019a). The statute provides that holding a cannabis license does not constitute an unsuitable act for purposes of Colorado’s gaming regulatory framework. When the bill was signed into law in 2019, it established the legal possibility for gaming operators to engage in cannabis business activities without violating state gaming laws.

Despite this permissive framework, the authors found no evidence that any Colorado gaming operator has entered the cannabis market. Several factors likely contribute to this outcome, including ongoing federal prohibition of cannabis, the extensive compliance requirements placed on gaming operators, and the potential reputational concerns associated with involvement in an industry still viewed as federally unlawful. Colorado’s experience illustrates that even in jurisdictions that allow cross-licensing, gaming operators may still avoid cannabis activities due to perceived federal and regulatory risks. This pattern aligns with the broader national trend in which gaming companies, including those operating in Colorado, continue to adopt a conservative approach to cannabis involvement. This dynamic is consistent with Nevada's central theme of regulatory stance and provides a valuable point of comparison for understanding why integration has not occurred, even where state law permits it.

### New Jersey

New Jersey has legalized both casino gaming and adult-use cannabis (N.J. Stat. § 5:12–119, 2024; N.J.A.C. 17:30, 2023). The State does not expressly prohibit gaming licensees from investing in cannabis businesses, apart from the multi-jurisdictional considerations discussed earlier. However, New Jersey maintains a separation between the two industries through P.L. 2021, c.025, which restricts cannabis consumption in public spaces, including casinos, and prohibits smoking or vaping cannabis on casino premises (New Jersey Cannabis Regulatory Commission 2022). In addition, although no statute or regulation directly addresses investment or ownership issues, the colocation of cannabis businesses on casino property is prohibited under state guidance and local regulatory practice (Danzis D 2020). Economic observers have noted that adult use of cannabis may contribute to broader revitalization efforts in Atlantic City, a region historically linked to gaming and tourism (Parmley 2023). While these discussions remain preliminary and are not the basis of current regulatory policy, they illustrate how cannabis activity may intersect with, but not necessarily integrate into, established gaming markets. For regulatory purposes, New Jersey continues to maintain a clear physical and operational separation between casino gaming and cannabis businesses, even within a permissive legalization framework.

### Rhode Island

As in Colorado and New Jersey, Rhode Island has no explicit statutory prohibition preventing gaming licensees from investing in the cannabis industry. However, the multi-jurisdictional nature of gaming licensing likely serves as a practical barrier to such involvement, since operators must maintain suitability in multiple states, including Nevada. This creates a strong disincentive for casinos to pursue cannabis related business activities even in the absence of a direct state-level restriction.

Rhode Island law reinforces this dynamic by specifying that casino operators are prohibited from engaging in conduct that is illegal in other jurisdictions (R.I.G.L. § 41–9.1–5, 2010; 230-RICR-30–30-2, 2022; Rhode Island Department of Business Regulation, (Rhode n.d). Although cannabis is legal under Rhode Island law, its continued illegality under federal law and in certain other states effectively prevents operators from integrating cannabis into casino operations or investing in cannabis businesses without risking broader licensing implications.

Rhode Island casinos also restrict cannabis consumption on their premises. While indoor use is not permitted, a spokesperson for Twin Rivers Casino reported that cannabis consumption would be allowed in designated outdoor areas (Bawden S 2022). This approach maintains compliance with state and local rules while acknowledging the legal status of cannabis in Rhode Island, yet it does not create any operational integration between the cannabis and gaming industries.

### Maryland

Maryland differs from New Jersey in that it does not expressly prohibit cannabis consumption in casinos. Instead, Maryland employs a form of reverse prohibition under Md. Code Ann., Alcoholic Beverages and Cannabis § 9–2705, which prevents cannabis licensees from hosting illegal gaming activities on their premises. While this provision primarily governs cannabis operators rather than gaming establishments, it effectively creates a boundary between the two industries by prohibiting cannabis businesses from engaging in activities associated with gaming.

As in other jurisdictions, the multi jurisdictional nature of gaming licensure likely discourages integration even without explicit prohibitions. Operators with interests in multiple states must maintain suitability in jurisdictions where cannabis remains federally prohibited or tightly regulated, which creates a strong incentive to avoid direct involvement with cannabis businesses.

Maryland law contains no statutory or regulatory restrictions that bar gaming operators from investing in cannabis businesses. However, available regulatory records and industry reports indicate that there have been no instances of Maryland gaming operators engaging in such investments (Maryland Gaming Control Agency). This absence suggests that, similar to trends observed in Colorado and New Jersey, operators remain cautious due to federal illegality and the broader regulatory environment rather than because of explicit state-level prohibitions (Maryland Gaming Control Agency 2025).

### The National Indian Gaming Commission (NIGC)

The National Indian Gaming Commission maintains a clear position regarding the presence of cannabis activities within tribal gaming facilities. Under NIGC guidance, if cannabis related conduct occurs inside a tribal gaming establishment, the Commission must refer the matter to federal law enforcement (NIGC, 2021). This requirement reflects the continued federal classification of cannabis as a Schedule I substance and underscores the legal constraints that tribal casinos encounter when evaluating potential involvement in cannabis related business activities.

The NIGC’s approach functions as an effective barrier to integrating cannabis operations with tribal gaming, even in states where cannabis is legal. It also parallels Nevada’s strict separation of the two industries by prioritizing compliance with federal law and protecting the integrity of gaming operations. Tribal casinos therefore face similar challenges to those in Nevada, where adherence to federal requirements and multi jurisdictional licensing norms shapes decisions that prevent integration, regardless of evolving state cannabis policies.

## Online gaming

An important consideration that has been largely absent from public discussions about separating the gaming and cannabis industries is the growth of online gaming and the ability for consumers to combine cannabis use with gaming activities in private settings (Nevada Legislature 2019a, b, c). Even in recent legislative testimony supporting the continued 1,500 foot separation requirement in Nevada, no references were made to the reality that online gaming allows individuals to engage in gaming and cannabis consumption simultaneously without entering a casino (Johnson 2018; Nevada Legislature 2025). Similarly, news coverage on the relationship between gaming and cannabis rarely mentions online gaming, aside from one casino executive who noted that it should be part of the broader policy conversation (Chouinard 2025; Wargo, 2025; Yakowicz 2025).

As stated earlier in this paper, the authors do not endorse the combination of cannabis use and gaming activities. There are valid public health concerns related to co-consumption and behavioral risks, which the existing literature highlights (Hammond et al. 2025; Horváth 2022; Dash 2019). As indicated, these issues generally fall outside the scope of the present analysis but remain important considerations for future research.

The purpose of this section is to examine gaps in regulatory and public discourse. Although statutory and regulatory frameworks impose strict physical and operational separation between cannabis businesses and brick-and-mortar gaming establishments, these policies do not address the increasingly common practice of consuming cannabis while participating in online gaming. This disconnect raises questions about the effectiveness and coherence of separation policies that focus solely on physical proximity. The growing prevalence of online gaming underscores the need for further study into the legal, regulatory, and public health implications of simultaneous cannabis use and online gaming, particularly as both industries continue to expand.

## Moving forward

Given the considerable time that has passed since the Nevada Gaming Control Board first instructed gaming licensees to avoid involvement in the cannabis industry, renewed discussion of the physical and regulatory barriers separating licensed cannabis and gaming activities is warranted. Federal policies have shifted, public attitudes toward cannabis have changed, and the broader legal landscape has evolved substantially since 2014. These developments warrant reassessing whether the original rationales for separation remain valid and whether the scope of current restrictions is consistent with contemporary regulatory conditions. Such reassessment should also address issues that were not fully examined when the separation was first adopted.

Although early guidance from several state regulators advised gaming licensees to refrain from participating in cannabis businesses, it may be useful to evaluate whether conditions exist under which limited engagement could occur before full federal legalization. For example, if major financial institutions and stock exchanges were to begin servicing cannabis businesses, or if federal enforcement priorities remained limited despite ongoing federal prohibition, it is unclear whether the gaming industry would continue to maintain a strict prohibition or reassess its position. Evaluating these questions depends on a clearer understanding of how regulators and operators assess federal enforcement risk and how that assessment influences licensing and compliance decisions.

Recent inquiries in other states highlight the need for clearer analysis. In 2023, the California Department of Cannabis Control requested guidance from the state Attorney General on whether interstate cannabis commerce might trigger federal enforcement (Lee, 2023). Nevada has not undertaken a similar assessment, nor has it evaluated which cannabis related activities might pose greater risk than others. For example, co-locating a consumption lounge on casino property may present different considerations than modifying the 1,500-foot setback requirement, which is not directly tied to federal cannabis enforcement.

Stakeholders in gaming jurisdictions may wish to consider whether existing prohibitions are broader than necessary or whether federal policy developments might permit a more nuanced approach. It may also be relevant to determine whether continued separation is justified if central financial institutions or national exchanges begin to support cannabis businesses before federal legalization occurs. Such developments could serve as indicators that cannabis related activities are viewed as carrying manageable federal risk.

Further analysis of the issues raised in this paper should include evaluating whether current distance setbacks and delivery prohibitions remain effective or necessary, assessing how cannabis business activities might affect the reputation of the gaming industry, determining whether licensed cannabis businesses could meet compliance standards comparable to those required in gaming, and identifying metrics that regulators can use to evaluate federal enforcement risk. Additional work is also needed to understand how multi-jurisdictional licensing could affect Nevada licensees if integration were permitted and to explore whether the two industries could collaborate on shared challenges, such as addressing unlicensed cannabis activity, without increasing exposure to federal enforcement. Addressing these questions will help clarify whether a revised approach to cannabis and gaming interactions could benefit both industries while maintaining regulatory integrity.

## Data Availability

No datasets were generated or analysed during the current study.
